# Supplementary material for: XRD Evaluation of Wurtzite Phase in MBE Grown Self-Catalyzed GaP Nanowires
Source: Nanomaterials (Basel). 2021 Apr 9;11(4):960. doi: 10.3390/nano11040960 (PMC8070561; doi:10.3390/nano11040960)
Supplement: Supplementary file 1 [file nanomaterials-11-00960-s001.pdf]

Supplementary Materials

XRD Evaluation of Wurtzite Phase in MBE Grown Self-Catalyzed GaP Nanowires

Olga Yu. Koval [1,*], Vladimir V. Fedorov [1,2], Alexey D. Bolshakov [1,3], Igor E. Eliseev [1], Sergey V. Fedina [1], Georgiy A. Sapunov [1], Stanislav A. Udovenko [2], Liliia N. Dvoretckaia [1], Demid A. Kirilenko [4], Roman G. Burkovsky [2] and Ivan S. Mukhin [1,3]

[1] Nanotechnology Research and Education Centre of the Russian Academy of Sciences, Alferov University, Khlopina 8/3, 194021 Saint Petersburg, Russia; burunduk.uk@gmail.com (V.V.F.); bolshakov@live.com (A.D.B.); eliseevie@gmail.com (I.E.E.); fedina.serg@yandex.ru (S.V.F.); sapunovgeorgiy@gmail.com (G.A.S.); liliyabutler@gmail.com (L.N.D); imukhin@yandex.ru (I.S.M.)

[2] Institute of Physics, Nanotechnology and Telecommunications, Peter the Great Saint Petersburg Polytechnic University, Politekhnicheskaya 29, 195251 Saint Petersburg, Russia; s_udovenko@mail.ru (S.A.U.); roman.burkovsky@gmail.com (R.G.B.)

[3] School of Photonics, ITMO University, Kronverksky Prospekt 49, 197101 Saint Petersburg, Russia

[4] Ioffe Institute, Politekhnicheskaya 26, 194021 Saint Petersburg, Russia; zumsisai@gmail.com

* Correspondence: o.yu.koval@gmail.com

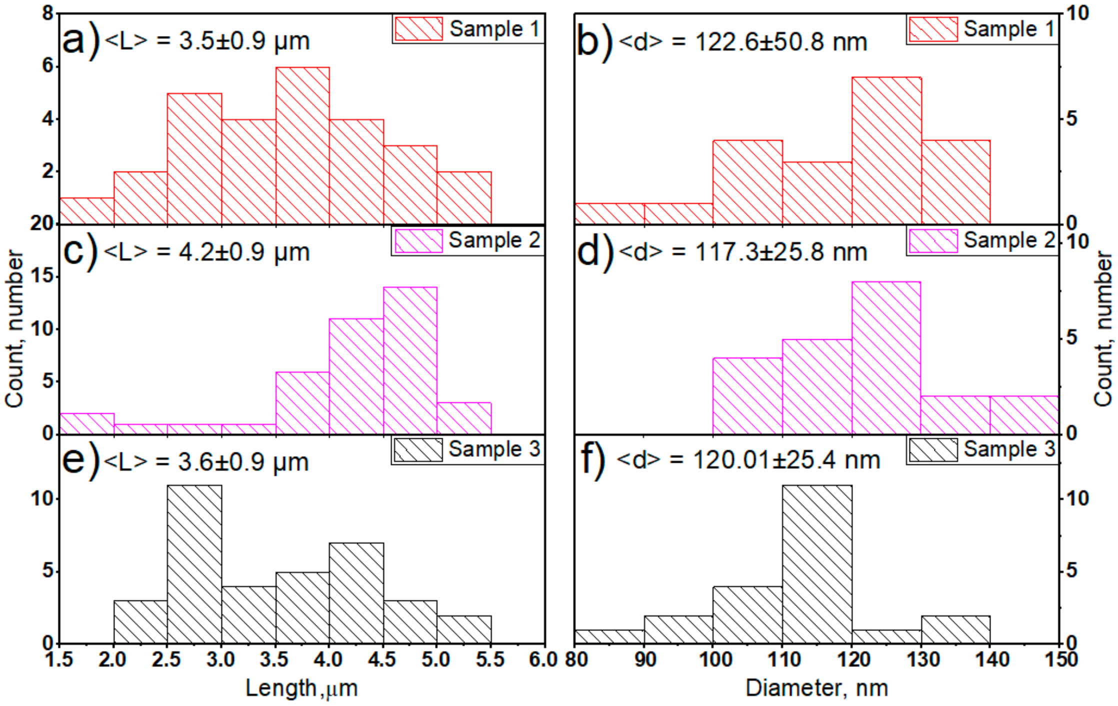

Figure S1. Length and diameter distributions of studied GaP NW arrays, where: (**a**,**b**)—Sample 1; (**c**,**d**)—Sample 2; (**e**,**f**)—Sample 3.

Length distributions were calculated using selection of ~30 NWs, Diameter distributions were calculated using selection of 22 NWs.

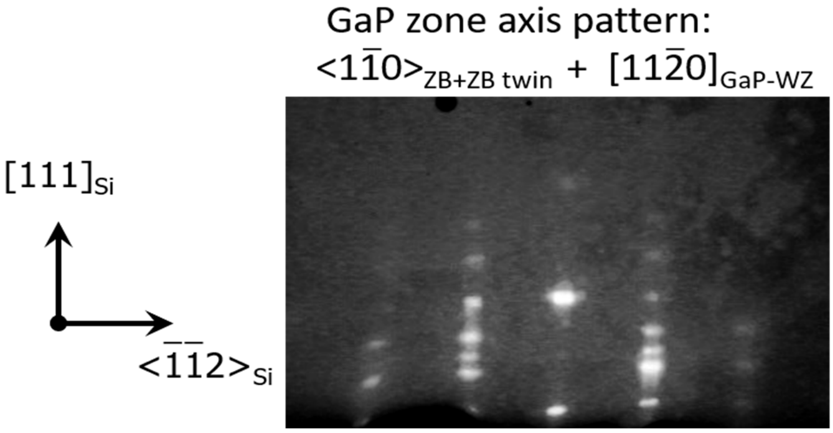

Figure S2. RHEED pattern of GaP NWs taken *in-situ* growth process.

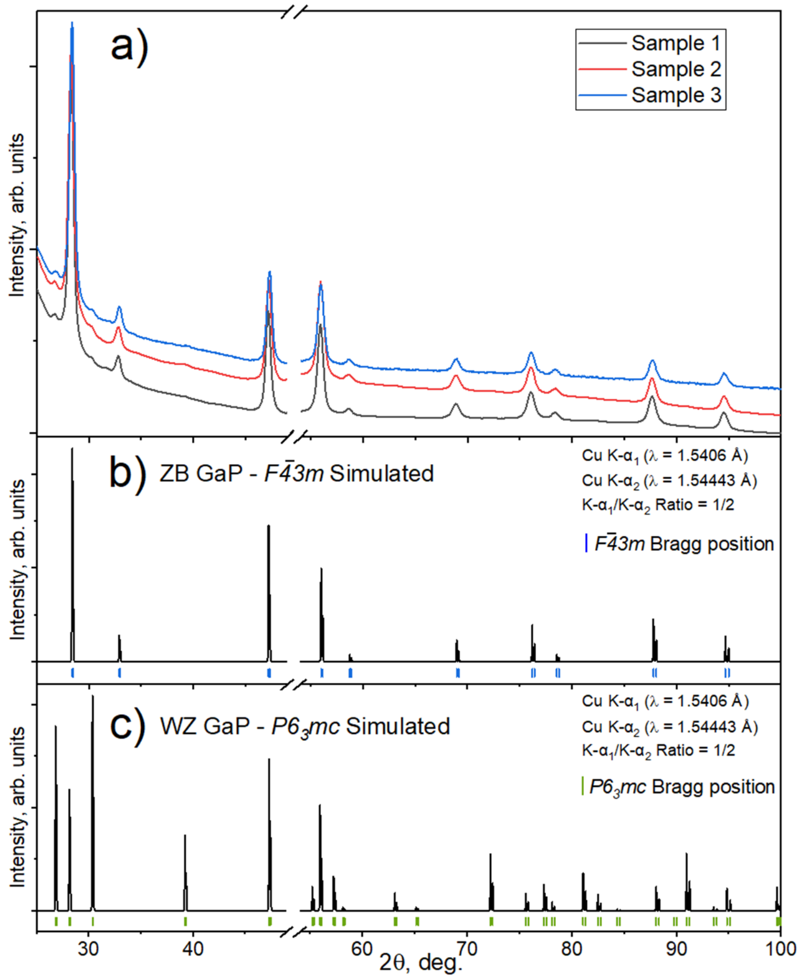

Figure S3. (**a**) Raw data before background subtraction of investigated GaP NW samples into scotch glue, (**b**) simulated XRD pattern for GaP ZB structure (**c**) simulated XRD pattern for GaP WZ structure. The tick marks correspond to the Bragg peak positions

Theoretical XRD presented in Figure S3b,c are model XRD, which were produced after refinement of lattice parameters for both ZB and WZ crystal phases for Cu Kα-radiation $\lambda = 1.5418$ Å. As clearly seen in Figure S3b,c several Bragg reflexes have the same position for these space groups of Gallium phosphide.

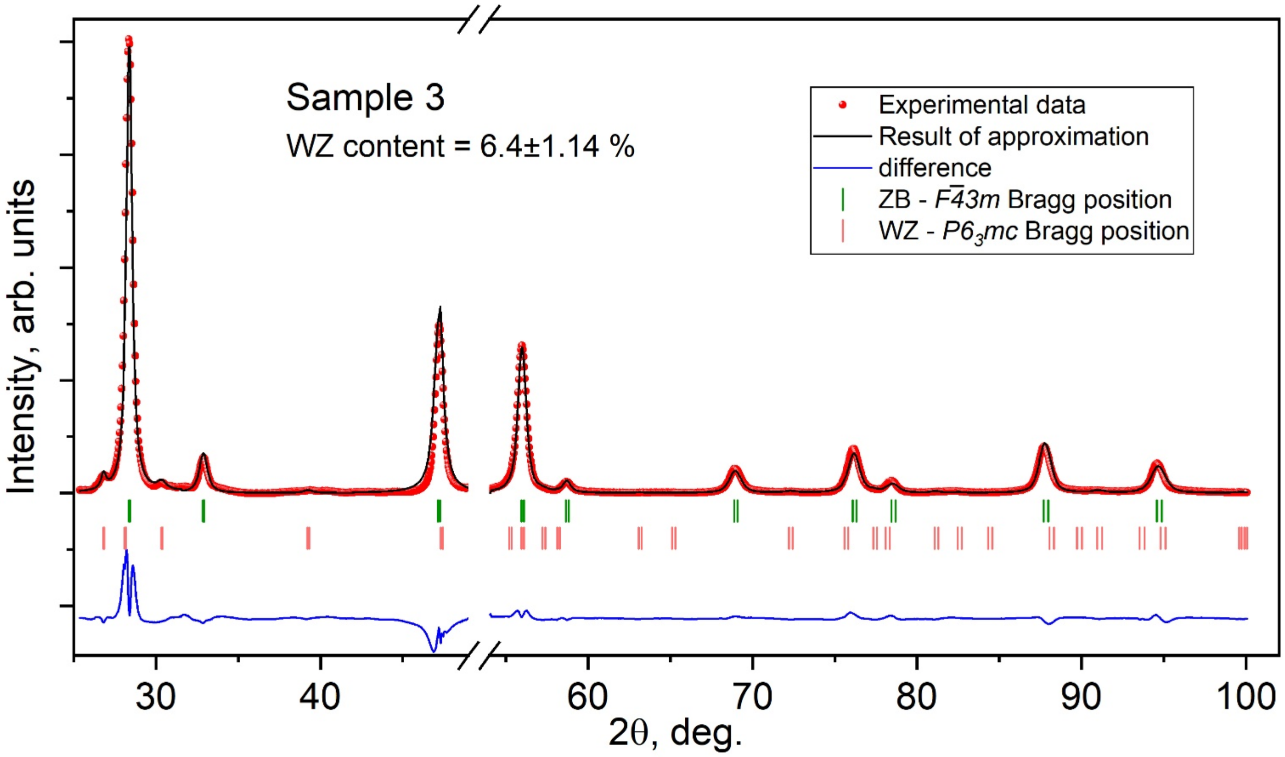

Figure S4. Approximated XRD powder diffraction pattern of NW array sample, which was grown at 620 °C (X-ray powder diffraction patterns were obtained using Cu K-alpha radiation $\lambda = 1.5418$ Å).
